# Living in the Pyrocene: Assemblage‐Wide Terrestrial Vertebrate Responses to Mega‐Fires in the Pantanal Wetlands

**DOI:** 10.1002/ece3.74130

**Published:** 2026-08-15

**Authors:** Natasha Grosch Loureiro, Diego Francis Passos Viana, Ciar D. Noble, Grasiela Edith de Oliveira Porfírio, Wener Hugo Arruda Moreno, Mariana Alves Carvalho Queiroz, Geovani Vinco Tonolli, Carlos A. Peres, Hugo Fernandes‐Ferreira

**Affiliations:** ^1^ Centro de Ciências, Departamento de Biologia, Programa de Pós‐graduação em Sistemática, Uso e Conservação da Biodiversidade Universidade Federal do Ceará Fortaleza Ceará Brazil; ^2^ Instituto Homem Pantaneiro ‐ IHP Corumbá MS Brazil; ^3^ School of Environmental Sciences University of East Anglia Norwich UK; ^4^ Instituto Juruá Manaus Amazonas Brazil; ^5^ Laboratório de Conservação de Vertebrados Terrestres (Converte) Universidade Estadual do Ceará Quixadá Ceará Brazil

**Keywords:** anthropogenic disturbance, forest‐dependence, habitat resilience, habitat selection, hierarchical modeling, mega‐fire

## Abstract

Fire is a natural evolutionary force that shapes ecosystems and population dynamics. However, recent increases in the frequency and severity of anthropogenic fires have had catastrophic impacts on biodiversity, even in fire‐prone environments. In Brazil's Pantanal Wetland biome, 3.9 million hectares were affected by fires in 2020, leading to the mortality of an estimated 17 million vertebrates. In the Serra do Amolar region, 61.2% of the extent of eight protected areas was burned. Here, we use multi‐year camera‐trapping sampling between 2019 and 2022 at 50 sites and hierarchical Bayesian models to investigate terrestrial vertebrate responses to wildfires across 35 species within the Pantanal's Serra do Amolar region. We did not find evidence of effects of wildfires on occupancy. Nonetheless, five mammal (capybara, ocelot, red brocket deer, jaguar, and lowland tapir), two bird (bare‐faced curassow and rufescent tiger‐heron), and two reptile species (common green iguana and tegu lizard) showed a positive association between their occurrence probability and forest cover, indicating their vulnerability under future scenarios of recurrent wildfires. Additionally, the vertebrate species composition differed over the study period. In 2022, four species (undulated tinamou, jaguarundi, crab‐eating raccoon, and Brazilian squirrel) were no longer detected. Among all species considered, 64% exhibited declines in relative abundance over time, with giant otters experiencing the most significant decline, while 36% showed increases. These findings raise significant concerns for wildlife conservation in the region, underscoring the need for long‐term monitoring and proactive management strategies to mitigate the impacts of recurring wildfires on species abundance. We also highlight the importance of Serra do Amolar as a potential wildlife refuge, considering species resilience to a novel fire regime.

## Graphical abstract

More than 60% of Serra do Amolar within the Pantanal Wetlands, Brazil, was burned in 2020. Nine vertebrate species showed a dependency on forest areas, which are more vulnerable to fires in this region, despite the fire not having shown a direct effect on the occupation. More than 60% of the evaluated species experienced a decline in abundance after the fire.

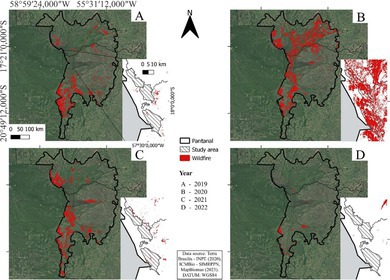

## 1 Introduction

Wildfires are an integral feature of the natural history of many fire‐prone ecosystems and have shaped the composition and functioning of resident biotic communities (Bowman et al. 2009; Nimmo et al. 2021). However, over recent decades, wildfire prevalence has rapidly increased throughout the world, likely due to increasing synergisms between land use and climate change (Gatti et al. 2021; Tyukavina et al. 2022; Abatzoglou et al. 2025). Fires now rage with greater severity and frequency, increasing biomass combustion and reducing recovery periods (Boer et al. 2016; Goss et al. 2020; Lindenmayer and Taylor 2020), with potentially catastrophic impacts on biodiversity. Indeed, changes in compounded disturbance regimes, including wildfires associated with habitat loss and fragmentation, have already led to a global‐scale decline in the abundance of ~60% of all terrestrial animals since the 1970s (Grooten and Almond 2018), and ~4400 species worldwide are under threat due to alterations in fire regimes (Kelly et al. 2020). Therefore, wildfires are currently considered a global problem for wildlife conservation and management (Hayward et al. 2016).

Life‐history traits influence species responses to habitat disturbance, including wildfires. Large‐bodied forest‐dependent species and dietary specialists tend to be more vulnerable to habitat change, while generalist herbivores may benefit from vegetation regrowth and reduced competition (Briani et al. 2004; Moretti and Legg 2009; Miller et al. 2019; Deus et al. 2023). Species dispersal capacity and adaptations to environmental filters, including fire return time, degree of seasonality, and fire severity and frequency, play a critical role in determining species persistence amidst mega‐fires, here defined as fires that affect more than one million hectares (Takach et al. 2022). Individuals that survive the direct impacts of fires tend to migrate to adjacent areas of the landscape that have not been affected, which is typically unfeasible in the face of extensive mega‐fires (Filkov et al. 2020). In addition to immediate lethal injuries, fires also induce animal mortality by starvation, due to the abrupt reduction in food resources, loss of habitat refugia, and opportunistic hyper‐predation (Filkov et al. 2020).

Unprecedented mega‐fires have recently devastated the Pantanal wetlands biome, affecting about 3.9 million hectares and reaching their peak in 2020 (INPE (Instituto Nacional de Pesquisas Espaciais) 2020; Libonati et al. 2020). In addition to structural damage to the ecosystem, it is estimated that around 17 million vertebrates died as a direct result of wildfires in the Pantanal in 2020, including nearly a million medium‐ and large‐bodied vertebrates (Tomas et al. 2021). Furthermore, approximately 65 million animals were impacted by habitat loss and resource shortage (Berlinck et al. 2022). In severely human‐modified landscapes, such as the Pantanal, the landscape is often characterized by heterogeneous habitat mosaics, which can influence patterns of habitat selection and species behavior (Karr and Freemark 1985; Johnstone et al. 2016). Such mosaics create spatial variation in resource availability, disturbance regimes, and structural complexity, altering the costs and benefits of habitat use. Species responses in terms of habitat selection depend on their degree of habitat specialization and the severity of disturbances experienced by those habitats (Elmqvist et al. 2003; Clavero et al. 2011).

For example, moist tropical forests are largely immune to wildfires, particularly at intervals larger than hundreds of years (Cochrane 2003). Yet these areas provide critical resources and refugia, particularly for larger‐bodied mammals and forest‐dependent taxa, potentially boosting survival, persistence, and even reestablishment in the aftermath of fire disturbance (Robinson et al. 2013). Shifting refuges are particularly important to highly seasonal ecosystems, such as the Pantanal wetlands, where fire and flood regimes are often critical stressors (Reside et al. 2019). Landscape heterogeneity creates breaks in the vegetation that, combined with other factors, such as the presence of large water bodies or water‐logged soils, provide buffer zones against fire (Robinson et al. 2013). Similarly, terrain elevation above the water table dictates microclimatic conditions and vegetation structure and influences patterns of habitat selection (Cordeiro and Neri 2019), which can be vital for species persistence in fire‐prone landscapes.

In this study, we seek to assess the assemblage‐wide dynamics of terrestrial vertebrates in the Serra do Amolar region, western border of the Brazilian Pantanal, in the face of recent recurrent mega‐fires. We examined the relative abundance of species across four consecutive years of sampling. We also examined the large‐scale influence of landscape features on habitat selection by these vertebrate species and further considered land cover and elevational gradients as key factors shaping habitat preference during and after wildfires. To do so, we assessed (1) the degree to which the local occupancy probability of different taxonomic groups (large‐bodied mammals, reptiles, and birds) in the terrestrial vertebrate assemblage of the western Pantanal is differentially affected by fractional burnt area, forest habitat, elevation, presence of domesticated species, and distance to water bodies; and (2) the changes in relative vertebrate abundance across all sampling periods. To answer these questions, we used hierarchical Bayesian multi‐species occupancy models, which ensure greater inferential robustness, while explicitly accounting for imperfect detection and the inclusion of scarce data from rare and cryptic species (Dorazio and Royle 2005; Dorazio et al. 2006).

## 2 Methods

### 2.1 Study Area

**FIGURE 1:**
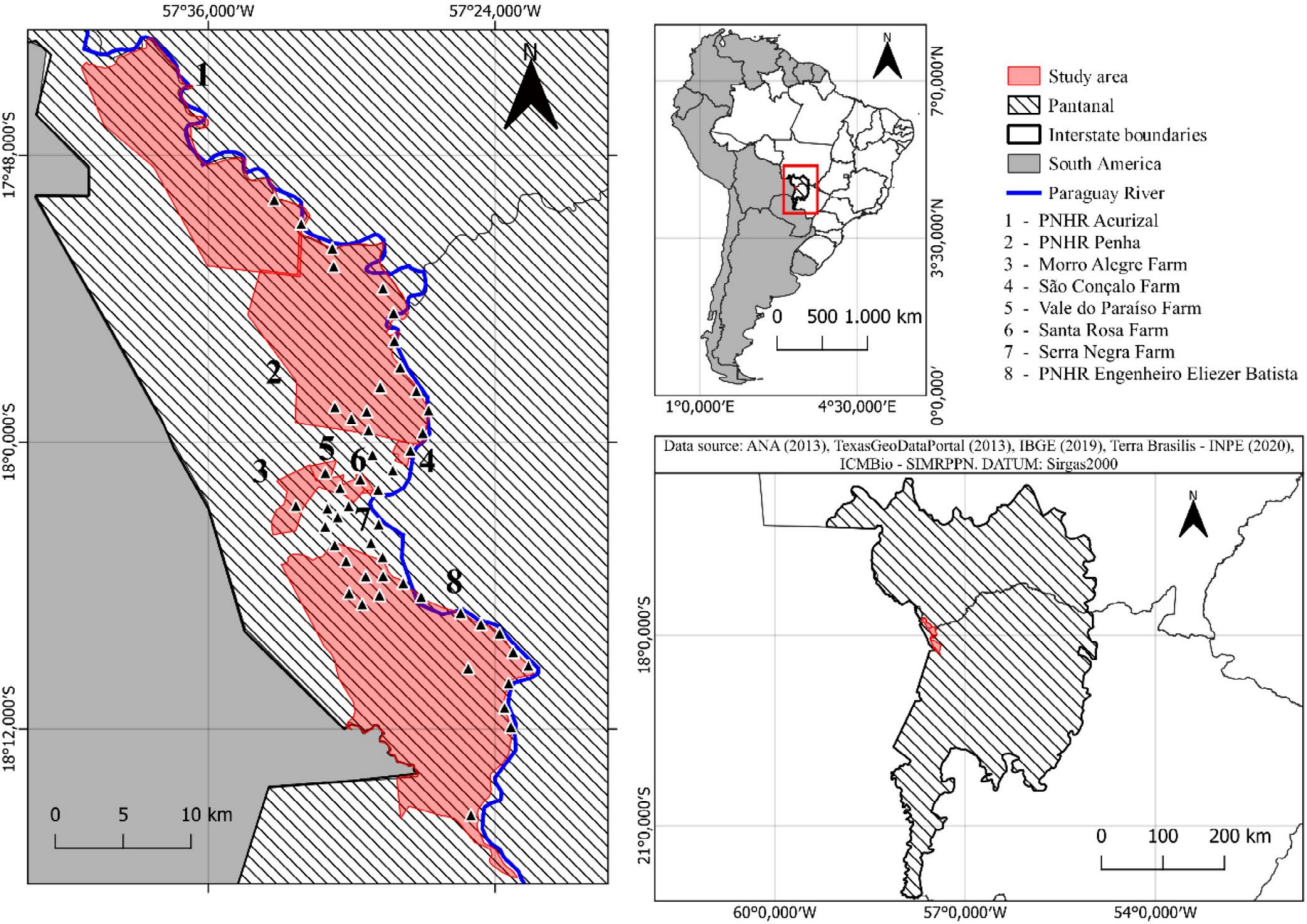
Location of the study area (in red) in the Pantanal wetlands biome within Brazil (hatched area) containing three formal protected areas [Private Natural Heritage Reserves (PNHR): Acurizal, Penha, and Engenheiro Eliezer Batista], in addition to five farm landholdings: Morro Alegre, São Gonçalo, Vale do Paraíso, Santa Rosa, and Serra Negra, and the sampling sites (black triangles). Camera‐trap stations were also deployed along the Paraguay River (blue line).

The ~210,000‐km^2^ Pantanal biome is the world's largest continental sheet‐flow wetland and covers parts of Brazil (62% of the entire Pantanal area), Bolivia (20%), and Paraguay (18%). Several protected areas are located within the Serra do Amolar region of the state of Mato Grosso do Sul, Brazil, within the western portion of the Pantanal wetlands. These include the 20,259‐ha Private Natural Heritage Reserves (PNHR) Engenheiro Eliezer Batista (18°05′25″ S, 57°28′24″ W), the 13,665‐ha PNHR Acurizal (17°49′51″ S, 57°33′06″ W), and the 13,490‐ha PNHR Penha (17°58′43″ S, 57°30′21″ W) (Figure 1). These areas are part of the Amolar Network, a complex of protected areas created by rural landowners (Porfirio et al. 2014). The Amolar region features a distinctive landscape characterized by an ecotone comprised of montane ranges, reaching elevations of up to 1000 m above sea‐level, and predominantly floodplain environments in the lowlands (Silva 2000; Junk et al. 2006). This unique topography is one of the reasons why the Amolar region was considered a priority area for biodiversity conservation in Brazil (MMA (Ministério do Meio Ambiente) 2018). According to the Köppen classification, the region's climate is characterized by hot, humid summers and cold, dry winters, with an average annual precipitation of 1300 mm (CADAVID‐Garcia 1984). The flood season occurs between May and June, while the dry season occurs between June and September, and the rainy season between October and April (Junk et al. 2006). In this region, fire activity is typically most intense when the Paraguay River recedes to its lowest water level. Fires usually begin in the uplands and gradually spread into the floodplains, but historically this was a rare, supra‐annual event with many years recording no evidence of fires (Damasceno‐Junior et al. 2021). In recent years, however, the anthropogenic fire regime has increased in terms of both severity and frequency (Marengo et al. 2021). In 2020, 61.2% of our study area (≈48,000 ha) burned, corresponding to approximately 29.000 ha. In addition, the vegetation of the region exhibits high levels of structural and compositional heterogeneity, including gallery and riparian forests, dry and humid savannas, semi‐deciduous and deciduous forests, and exposed bedrock areas (SÁ Arruda et al. 2012).

### 2.2 Vertebrate Sampling

We conducted sampling at 50 sites (within the PNHRs and along the Paraguay River) within a camera‐trapping grid, in which traps were spaced apart by at least 1 km (Figure 1). At each sampling point, one or two paired camera traps were installed. The data collection campaigns took place each year between 2019 and 2022, spaced across three sampling periods: from December 2019 to February 2020, spanning a period before the major western Pantanal fires of 2020; and two periods in the aftermath of major fires, from December 2020 to February 2021 and from February to April 2022. Individual camera trap stations were active for an average sampling effort of 41 nights, ranging from 4 to 109 consecutive nights, across the entire study period. This resulted in a total sampling effort of 8295 camera‐trap‐nights (hereafter, CTNs = number of cameras × number of exposure days/nights). To determine the detection independence between consecutive photographic records of each species, we considered a minimum interval of 30 min between records (O'Brien et al. 2003). Only records of 35 detectable mammals, reptiles, and terrestrial bird species were retained, thereby excluding records of 28 arboreal and volant species (i.e., species that are not usually targeted by camera traps installed close to the ground). We also excluded all species detected at only one site, which were considered to be occasional.

### 2.3 Explanatory Variables

We defined five covariates to model patterns of species' occurrences: (1) the proportional area burnt within a series of radial buffers (i.e., 100, 500, 1000, and 2000 m) around each camera‐trap station, which was obtained from fire monitoring data by the MapBiomas project (Fogo v. 2; MAPBIOMAS 2023a). These spatial data have a 30‐m resolution and originate from the Landsat time series. For analysis, we computed the fire data accumulated over 12 months before the first month of each sampling period (i.e., if any cell was burned in the preceding 12 months it was assigned a value of 1, or otherwise, 0; Figure S1). (2) The percentage of forest cover within a buffer (i.e., 100, 500, 1000, and 2000 m) was obtained from the MapBiomas project v. 8.0 (MAPBIOMAS 2023b). These spatial data have a 30‐m resolution and were obtained from Landsat‐8 satellite imagery. We quantified forest cover within the buffers according to the year in which sampling began at each site and sampling period (Figure S2). (3) Elevation at each sampling point, extracted from the 30‐m resolution SRTM (Shuttle Radar Topography Mission) digital elevation model obtained from the U.S. Geological Survey (USGS (U.S. Geological Survey) 2023) (Figure S3). (4) Semi‐domesticated animals (i.e., cattle, domestic dogs, horses, and pigs) and human presence, which were grouped in the camera‐trapping database (i.e., records in images of all these species were assigned to the same category). (5) Minimum distance to the nearest water body, calculated according to water body polygons obtained from the Global Water Surface platform (Pekel et al. 2016; Figure S4). As the exact scale at which these factors influence species occurrence probabilities was not known and may vary considerably among taxa, we extracted metric values from within a series of variable‐sized radial buffers (100, 500, 1000, and 2000 m) and then used sensitivity analyses to determine the optimal buffer sizes for each vertebrate class (see Section 2.4.2 for details).

For each sampling point in each sampling period (week), we also extracted four variables that could influence species' detectability: (1) Daily precipitation (mm/day), obtained from the NASA POWER (National Aeronautics and Space Administration—Prediction of Worldwide Energy Resource) platform, derived from Global Precipitation Measurement (GPM) mission's via Integrated Multi‐satellitE Retrievals for GPM (IMERG) (NASA/POWER (National Aeronautics and Space Administration ‐ Prediction of Worldwide Energy Resource) 2023). (2) Mean daily temperature (°C) was also obtained from the same platform derived from NASA's GMAO MERRA‐2 and GEOS (Goddard Earth Observing System) 5.12.4 FP‐IT (NASA/POWER (National Aeronautics and Space Administration ‐ Prediction of Worldwide Energy Resource) 2023). Finally, we considered (3) the number of camera traps operating at each sampling site in each sampling period; and (4) the number of whole diurnal periods in which each camera trap had been operated during each occasion (1 to 7 days).

To examine correlations among these covariates and among the detection covariates, we derived Pearson's Correlation Coefficients (Dormann et al. 2012). Significant correlations (r ≥ |0.7|) were found only between different buffer sizes of the same variables (Figure S5). As only one buffer size was used for each model, all variables were retained. We applied multiple buffer sizes to identify the ‘best’ scale of effect showing the strongest predictive power of landscape scale covariates (see below).

### 2.4 Statistical Analysis

#### 2.4.1 Relative Abundance

The raw relative abundance (i.e., records per 100 CTNs) was calculated by dividing each species' total number of records by the effort (CTN) and multiplying by 100. CTNs were calculated for each station during each sampling period. Abundance was compared across the three sampling periods (see above in 2.2 Vertebrate sampling), both before and after the 2020 M‐fire event. Only species that had been recorded during all three sampling periods were considered in this analysis, to avoid bias arising from differences in species' detection frequency and habitat use across periods.

#### 2.4.2 Multi‐Species Occupancy Modeling

We modeled species occurrence and detection probabilities using a Bayesian hierarchical multi‐season, multi‐species occupancy model. This enabled us to quantify the impacts of wildfires, land cover, and elevation on the occurrence of terrestrial vertebrate species while controlling for imperfect detection in our sampling (Dorazio and Royle 2005; Dorazio et al. 2006; Doser et al. 2022). We fitted our model to a binary detection/non‐detection array $Y_{i,j,p,k}$ with elements indicating whether each species i was detected within sampling period k (i.e., seven consecutive camera trapping days) at site j in year p. We considered $Y_{i,j,p,k}$ to be an imperfect estimate of the true (latent) occurrence states $z_{i,j,p}$.

To control for spatial autocorrelation in species occurrence probabilities among sites, we also included a species‐specific Nearest Neighbor Gaussian Processes in the occurrence model component (Datta et al. 2016), considering the three nearest sampling sites to each camera trap station as neighbors. The model likelihood thus took the general form:

$$\begin{array}{c} y_{i,j,p,k}\sim \text{Bernoulli}\left(\theta_{i,j,p,k}\times z_{i,j,p}\right)\\ z_{i,j,p}\sim \text{Bernoulli}\left(\psi_{i,j,p}\right)\\ \text{Logit}\left(\psi_{i,j,p}\right)=\alpha_{\psi ,i}+\eta_{i,j,p}+\beta_{\psi ,i}X_{\psi ,j,p}\\ \text{Logit}\left(\theta_{i,j,p,k}\right)=\alpha_{\theta ,i}+\beta_{\theta ,i}X_{\theta ,j,p,k} \end{array}$$

where $\theta_{i,j,p,k}$ is the probability of detecting species i at site j in year p during sampling period k; $\psi_{i,j,p}$ is the probability that species i truly occurred at site j in year p; $\alpha_{\psi ,i}$ and $\alpha_{\theta ,i}$ represent the species‐specific occurrence and detection model intercepts, respectively; $\beta_{\psi ,i}$ and $\beta_{\theta ,i}$ represent the species‐specific occurrence and detection model slopes, respectively; $X_{\psi ,j,p}$ and $X_{\theta ,j,p,k}$ are fixed‐effects design matrices; and $\eta_{i,j,p}$ represents the species‐specific spatial random effect on occurrence.

All species‐specific coefficients (α, β) were drawn from community‐level hyperparameter distributions (Dorazio et al. 2006). These hyperparameters thus represent the full distribution of species‐specific covariate responses within the community. We specified weakly informative priors on all hyperparameter components. Specifically, we used Normal (0, 2.72) priors for all hyperparameter means, and Inverse‐Gamma (0.1, 0.1) for all hyperparameter variances (τ^2^β and τ^2^α).

Inference was performed using four Markov Chain Monte Carlo (MCMC) chains of 150,000 iterations, each with a “burn‐in” of 10,000 iterations and a thinning rate of 10. Model convergence was assessed using the Gelman‐Rubin Diagnostic ($\hat{R}$), where values below 1.1 indicated satisfactory convergence (Gelman and Rubin 1992). To determine the optimal structure of the detection model, we first modeled detection probability independently, using a null occupancy model (i.e., including $\alpha_{\psi ,i}$ and $\eta_{i,j,p}$ only). We fitted models including all possible combinations of detection covariates and then ranked these models based on their Watanabe‐Akaike Information Criterion (WAIC). We considered the model with the lowest WAIC score to be the best performing and kept this constant for all further analyses (Watanabe 2010; Gelman et al. 2013; Table S1).

To determine the most suitable buffer size (i.e., 100, 500, 1000, or 2000 m) for each variable (i.e., percentage of burned area and land use and land cover classes) and taxa (i.e., birds, mammals, and reptiles), a model selection was also performed using WAIC (Watanabe 2010; Gelman et al. 2013; Table S2). The most parsimonious models (i.e., those with the lowest WAIC scores) included buffers of 1000 m for mammals, 2000 m for birds, and 100 m for reptiles (Table S2). All analysis were performed using the software R (R Core Team 2023).

## 3 Results

From a total of 2674 wildlife records, we detected 35 vertebrate species across the study area at all sampling stations (naïve species richness), including one Endangered species ( *Pteronura brasiliensis* ), five Vulnerable species ( *Crax fasciolata* , *Myrmecophaga tridactyla* , *Priodontes maximus* , *Tapirus terrestris*, and *Tayassu pecari* ), one Near‐Threatened species ( *Panthera onca* ), 26 Least‐Concern species, and two Data Deficient species ( *Mazama americana* and *Dasyprocta azarae* ) according to the IUCN (Table 1). In the final year of data collection (2022), four species were no longer detected ( *Crypturellus undulatus* , *Herpailurus yagouaroundi*, *Procyon cancrivorus* , and *Guerlinguetus aestuans*). The three most frequently recorded species were *C. fasciolata* with 526 records, *D. azarae* with 383 records, and *Hydrochoerus hydrochaeris* with 302 records.

**TABLE 1:** Checklist of the 35 vertebrate species detected in the Western Pantanal throughout the sampling period (between 2019 and 2022) in this study, taxa (birds, mammals, and reptiles), and their respective levels of threat according to the International Union for Conservation of Nature (IUCN). LC: Least Concern; DD: Data Deficient; NT: Near Threatened; VU: Vulnerable; and EN: Endangered. Rows marked in bold show threatened species.

Common name	Species name	Taxa	IUCN classification
Blue‐throated Piping‐guan	*Aburria cumanensis*	Bird	LC
Gray‐cowled Wood‐rail	*Aramides cajaneus*	Bird	LC
Cocoi Heron	*Ardea cocoi*	Bird	LC
Red‐legged Seriema	*Cariama cristata*	Bird	LC
Bare‐faced Curassow	*Crax fasciolata*	Bird	VU
Undulated Tinamou	*Crypturellus undulatus*	Bird	LC
White‐tipped Dove	*Leptotila verreauxi*	Bird	LC
Chaco Chachalaca	*Ortalis canicollis*	Bird	LC
Buff‐necked Ibis	*Theristicus caudatus*	Bird	LC
Rufescent Tiger‐heron	*Tigrisoma lineatum*	Bird	LC
Crab‐eating Fox	*Cerdocyon thous*	Mammal	LC
Agouti	*Dasyprocta azarae*	Mammal	DD
Nine‐banded Armadillo	*Dasypus novemcinctus*	Mammal	LC
Collared Peccary	*Dicotyles tajacu*	Mammal	LC
White‐eared Opossum	*Didelphis albiventris*	Mammal	LC
Tayra	*Eira barbara*	Mammal	LC
Yellow Armadillo	*Euphractus sexcinctus*	Mammal	LC
Brazilian squirrel	*Guerlinguetus aestuans*	Mammal	LC
Jaguarundi	*Herpailurus yagouaroundi*	Mammal	LC
Capybara	*Hydrochoerus hydrochaeris*	Mammal	LC
Ocelot	*Leopardus pardalis*	Mammal	LC
Red Brocket deer	*Mazama americana*	Mammal	DD
Giant Anteater	*Myrmecophaga tridactyla*	Mammal	VU
South American Coati	*Nasua nasua*	Mammal	LC
Jaguar	*Panthera onca*	Mammal	NT
Giant Armadillo	*Priodontes maximus*	Mammal	VU
Crab‐eating Raccoon	*Procyon cancrivorus*	Mammal	LC
Giant Otter	*Pteronura brasiliensis*	Mammal	EN
Puma	*Puma concolor*	Mammal	LC
Gray Brocket deer	*Subulo gouazoubira*	Mammal	LC
Lowland Tapir	*Tapirus terrestris*	Mammal	VU
White‐lipped Peccary	*Tayassu pecari*	Mammal	VU
Giant Ameiva	*Ameiva ameiva*	Reptile	LC
Common Green Iguana	*Iguana iguana*	Reptile	LC
Tegu	*Tupinambis* sp	Reptile	LC

### 3.1 Relative Abundance

**FIGURE 2:**
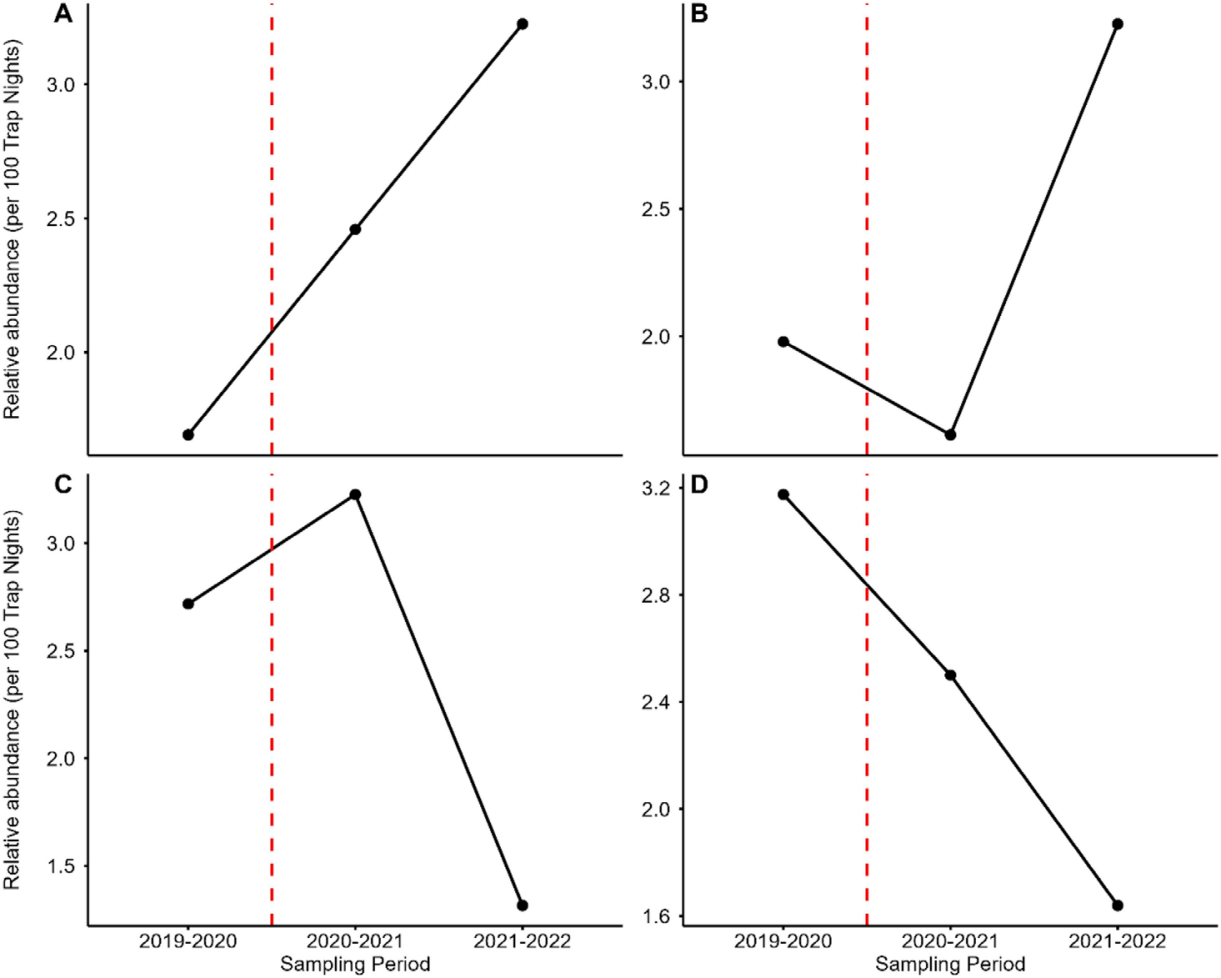
Raw relative abundance (i.e., independent photos per 100 CTNs) over the three sampling periods before the major western Pantanal fires (2019–20) and in the aftermath of major fires (2020–21 and 2021–22). The red dashed line represents the 2020 megafire event. The panels display species plots as follows: A = *Ameiva ameiva* , B = *Dasypus novemcinctus* , C = *Pteronura brasiliensis* , and D = *Ardea cocoi* .

Among all 35 species, 25 were recorded during all sampling periods. Most species (64%) exhibited a decline in their relative abundance between the first sampling period before the 2020 fires and the final period in 2022 in the aftermath of those fires. However, nine species (36%) showed an overall increase between these two sampling periods (Figures 2, S6 and S7, Table S3). The giant Ameiva ( *A. ameiva* ) exhibited the greatest increase in naïve abundance (90.7%), followed by nine‐banded armadillo ( *D. novemcinctus* ; 63.1%), while the species showing the steepest decline was the giant otter ( *P. brasiliensis* ; 51.6%), followed by the rufescent tiger‐heron, *A. cocoi* (48.4%) (Figures 2 and S8, Table S3).

### 3.2 Assemblage‐Level Predictors of Occupancy

**FIGURE 3:**
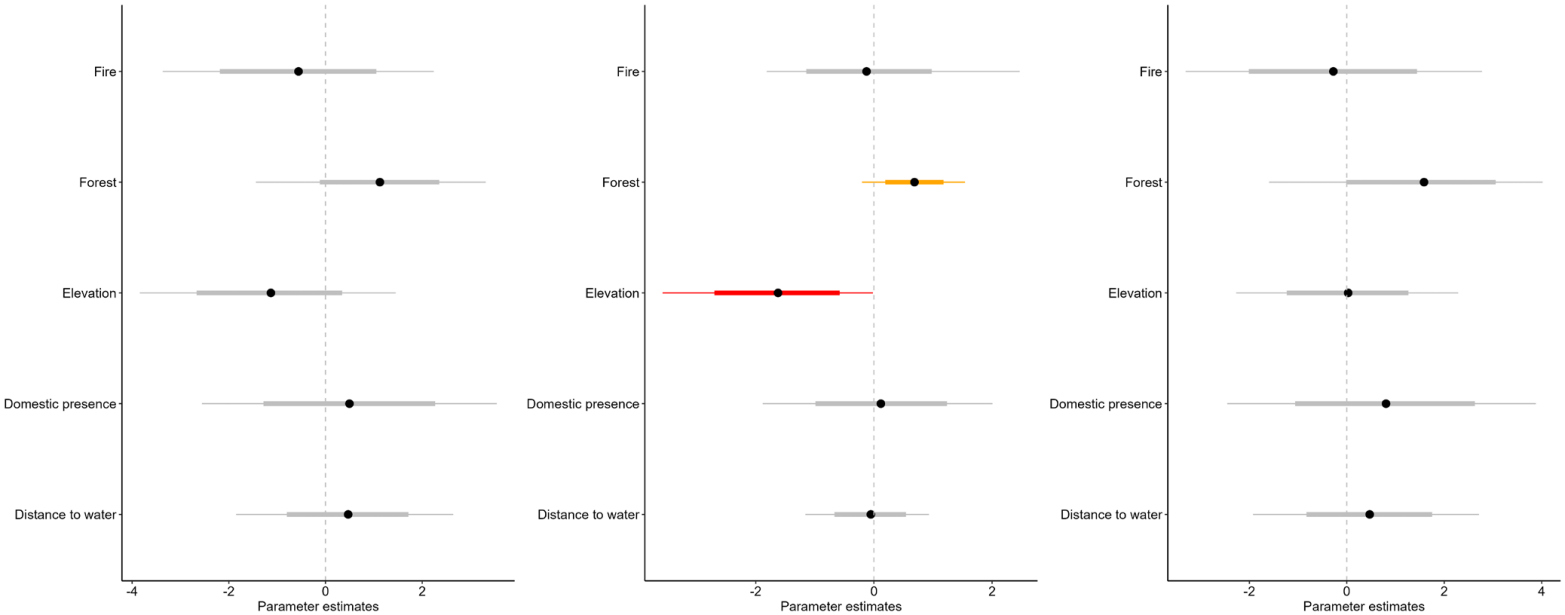
Mean beta coefficients for assemblage‐level vertebrate occupancy between 2019 and 2022 at Serra do Amolar, Mato Grosso do Sul, Brazil, embedded within the Pantanal wetlands. Gray bars represent relationships for which the 75% Bayesian credible intervals (BCIs) include zero. Orange bars indicate that the 75% BCI excludes zero. Red bars represent negative relationships, in which the 95% BCI excludes zero. Panels display effect sizes for bird, mammal, and reptile assemblages from left to right.

At the mammal community‐level, forest areas positively affected overall occupancy, while elevation exerted a negative effect (Figure 3). However, the positive forest area effect was only moderately supported and detected only at the 75% BCI level (posterior probabilities that did not overlap zero), while the negative effect of elevation was strongly supported at the 95% BCI level. For reptiles and birds, none of the predictor variables influenced patterns of occupancy probability (Figure 3). Fire didn't show any effect at the community level.

### 3.3 Species‐Level Predictors of Occupancy

**FIGURE 4:**
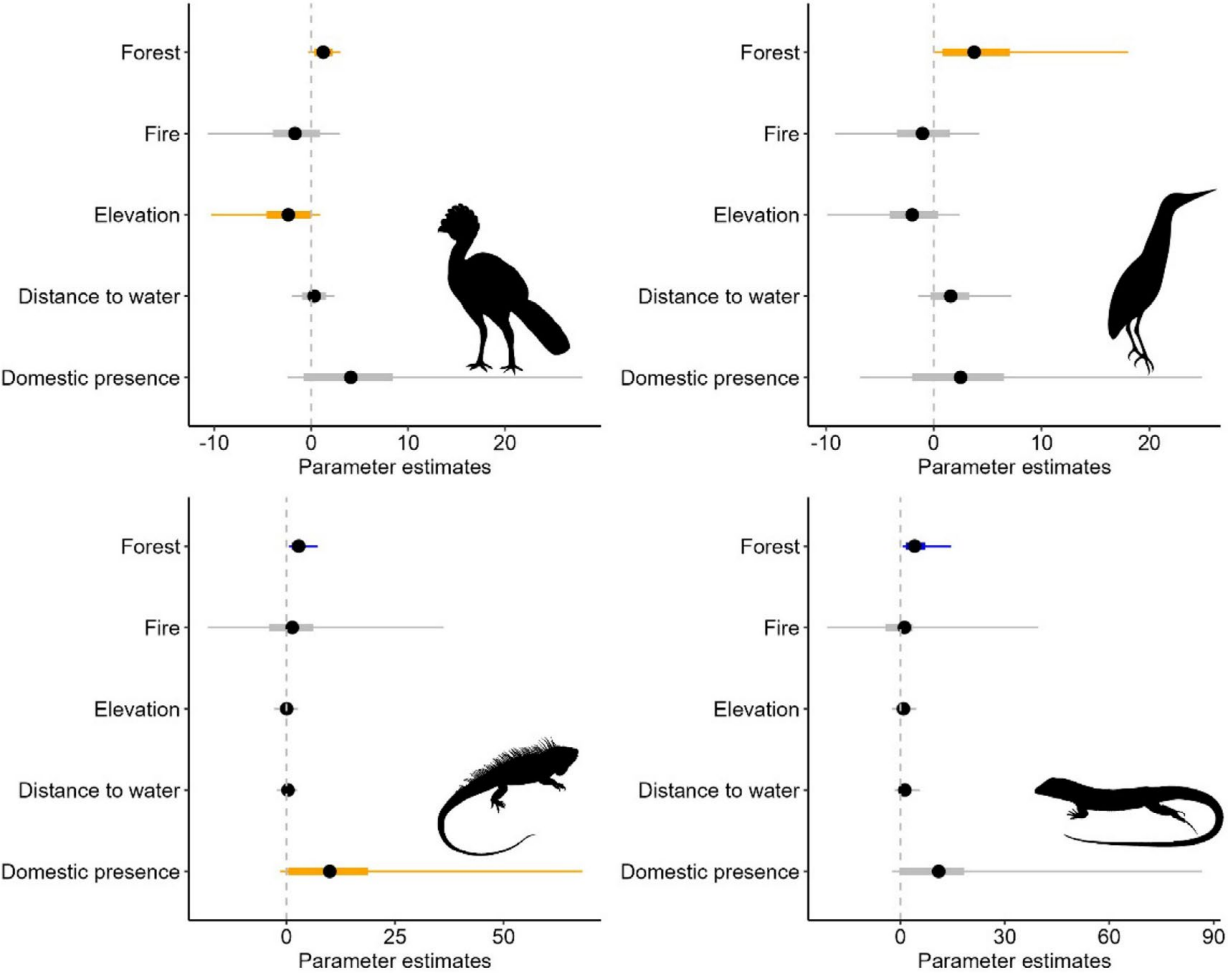
Mean beta coefficients for species‐level occupancy between 2019 and 2022 at Serra do Amolar, Mato Grosso do Sul, Brazil, embedded within the Pantanal wetlands. Gray bars represent relationships where the 75% Bayesian credible intervals (BCIs) include zero. Orange bars indicate that the 75% BCI excludes zero, while the 95% BCI still includes zero. Blue bars represent positive relationships, which the 95% BCI excludes zero. The panels display graphs for *Crax fasciolata*, *Tigrisoma lineatum* , *Iguana iguana* , and *Tupinambis* sp. from left to right and top to bottom. Images from PhyloPic.org were used under Creative Commons licenses. Silhouettes were created for *Crax fasciolata* by Gabriela Palomo‐Munoz (CC BY 3.0), *Iguana iguana* by Jack Mayer Wood (CC BY 1.0), and *Tupinambis* sp. by Jose Carlos Arenas‐Monroy (CC BY 1.0). For *Tigrisoma lineatum* silhouette was created based on an illustration used with permission from Asociación Armonía/Birds of Bolivia—Field Guide.

Among all 10 bird species, environmental variables significantly affected the occupancy of only bare‐faced curassow ( *Crax fasciolata* ) and rufescent tiger‐heron ( *Tigrisoma lineatum* ) at the 75% BCI level. The former was negatively associated with elevation and positively affected by forests, while the latter was only positively affected by forested areas (Figures 4, S8, and S9).

For reptiles, the occupancy of two of the three species was significantly influenced by at least some predictors (Figures 4, S8, and S9). The occurrence of *Iguana iguana* was positively influenced by forest areas and the presence of humans and domesticated animals (Figure 4), while *Tupinambis* sp. was only positively affected by forest areas (Figure 4).

**FIGURE 5:**
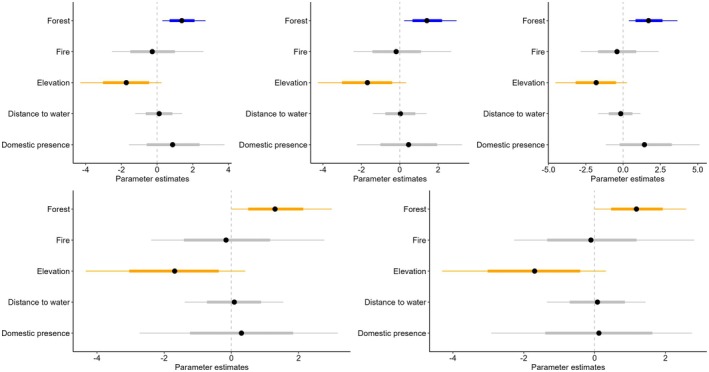
Mean beta coefficients for species‐level occupancy between 2019 and 2022 at Serra do Amolar, Mato Grosso do Sul, Brazil, embedded within the Pantanal wetlands. Gray bars represent relationships where the 75% Bayesian credible intervals (BCIs) include zero. Orange bars indicate that the 75% BCI excludes zero, while the 95% BCI still includes zero. Blue bars represent positive relationships, which the 95% BCI excludes zero. The panels display graphs for *Hydrochoerus hydrochaeris* , *Leopardus pardalis* , *Panthera onca, Mazama americana*, and *Tapirus terrestris* , from left to right and top to bottom.

For mammals, occupancy of all species was affected by at least one environmental variable. All 22 species were negatively impacted by elevation (at the 75% BCI level). Additionally, *Hydrochoerus hydrochaeris* , *Leopardus pardalis* , and *Panthera onca* were positively influenced by forest areas (at the 95% BCIs level), which also positively influenced *Mazama americana* and *Tapirus terrestris* at the 75% BCI level (Figures 5, S8, and S10). Fire didn't show any effect at the species level.

## 4 Discussion

Our findings suggest that the area burned between 2019 and 2022 did not have a direct effect on vertebrate species' probability of occupancy at the Serra do Amolar landscape. However, shifts in species composition were evident by the final sampling period, with the disappearance of four species. Additionally, two‐thirds of all species exhibited a decline in relative abundance, indicating that fire‐induced habitat changes may be influencing species persistence over time. Also, forest cover, which can be devastated by wildfires (Correa et al. 2022; Tyukavina et al. 2022), emerged as a significant positive determinant of the occupancy of several species.

Although the distance to open water did not significantly influence species occupancy, 66% (33 out of 50) of our camera‐trap stations were within 100 m of water bodies, and the longest distance was less than 3 km, which means that we sampled these areas more, and these areas may have served as refugia. In fire‐prone environments, many species seek shelter in water bodies (Nimmo et al. 2021), which help mitigate fire impacts by providing escape routes, reducing thermal stress, and lowering immediate mortality. The high humidity and moist biomass in these areas can slow or even prevent fire spread, acting as natural buffer zones and above‐ground refuges (Pettit and Naiman 2007; Rojas et al. 2022), particularly for non‐fossorial species. Additionally, these areas provide alternative food sources, which may explain why vertebrate occupancy did not decline as dramatically as expected. However, with increasingly severe droughts predicted for the Pantanal, these critical refugia may shrink, further threatening the long‐term persistence of the region's vertebrate fauna (Marengo et al. 2021; Costa et al. 2024).

While the wildfires did not directly influence habitat selection at the community or species level, the spatial use of five rodents, ungulate, and carnivore species ( *Hydrochoerus hydrochaeris* , *Leopardus pardalis* , *Mazama americana* , *Panthera onca* , and *Tapirus terrestris* ) and four birds and reptiles showed a clear preference for forested areas. Given the increasing frequency and severity of wildfires (Pelissari et al. 2023), these late‐successional habitats are likely to face ongoing degradation. Their slow regeneration rates contrast with the rapid colonization of fire‐adapted pioneer species like grasses (Issifu et al. 2021). After the episodic wildfires and the subsequent flooding, some of the colonizing species in the postburn recruitment are also lost (Oliveira et al. 2014), further hindering forest regeneration dynamics. In ecosystems affected by frequent fires and floods, a long‐term trend is the persistence of open areas dominated by a limited number of tree species (Pott et al. 2011; Silva et al. 2020; Cunha et al. 2022). Furthermore, although wildfires over the past two decades in the Pantanal wetlands predominantly affected grassland, the 2020 fires caused the most significant damage to forested areas (Correa et al. 2022). As a result, wildfires may indirectly alter habitat selection and contribute to habitat loss for forest‐dependent species, particularly many threatened vertebrate species typical of forest domains elsewhere, which rely on tree cover in advanced successional stages (Sales et al. 2020; Takach et al. 2022). The impact of mega‐fires on vertebrate communities is well‐documented globally. In Australia, Ward et al. (2020) reported that the 2019–2020 wildfires led to habitat loss for one‐third of assessed species, with some losing up to 30% of their habitat. Their findings underscore the crucial role of unburned refugia in mitigating long‐term biodiversity loss, a factor that may also influence species persistence in the Pantanal. Moreover, some of these vertebrates exhibit low‐fecundity traits, such as delayed sexual maturity, prolonged gestation, small litter sizes, long inter‐birth intervals, and extended parental care, all of which require additional time and resources to ensure generational replacement (Gatti et al. 2011; Mayor et al. 2011; Ortiz et al. 2022). This further exacerbates concerns regarding their long‐term conservation.

The apparent lack of fire effects on occupancy may also be related to the time elapsed since the fire (three months after the peak of fire outbreaks) when repeated sampling was conducted, as several transient effects are typically observable only immediately following fire events (Batista et al. 2023). Other changes may take longer to manifest. Evidence of delayed mortality or reduced survival may not be detected until years or even decades later, as habitat structure and key trophic resources degrade over time (Sherman and Runge 2002; Arthur et al. 2012). The methodological differences between our study and Tomas et al. (2021) likely account for the contrasting findings, and also consider their extrapolation from their study area to the entire biome. While their study estimated ~17 million vertebrate deaths using carcass surveys (capturing immediate postfire mortality), our camera‐trap study primarily recorded surviving individuals. This discrepancy highlights the importance of employing multi‐method approaches to fully assess the impacts of fire on wildlife.

The chronosequence at Serra do Amolar aligns with other studies that also failed to detect significant direct negative effects of fire on the vertebrate fauna. Deus et al. (2023) found no evidence of fire impacting mammalian diversity in the northern Pantanal, while Bardales et al. (2024) reported no influence of fire on the colonization and extinction dynamics of six mammals in the Serra do Amolar but found an increase in habitat use of *Panthera onca* (suggesting movement from severely affected forests to riparian forest with quick regeneration) and a low rate of individuals recaptured, indicating mortality of individuals from the territory or population flow (immigration of individuals) sustaining the population. In contrast, using camera trapping associated with eDNA, Magioli et al. (2024) observed increased mammal species richness one year after wildfires in the northern Pantanal, likely due to the immigration of herbivorous species exploiting the new flush of vegetation in postburn open areas. Furthermore, while they found differences in species composition between burned and unburned areas, there were no significant variations in species richness or abundance. Eriksson et al. (2025) also reported evidence of a potential climate refuge in northern Pantanal, where mammal richness increased across their study area following the 2020 wildfires, showing resilience. This indicates that, despite climate‐driven disturbances, the community of medium and large mammals can show notable resilience when suitable refuges remain available.

Mammals and bare‐faced curassow also responded negatively in their probability of occurrence with increasing terrain elevation. The altitudinal gradient pattern and the mechanistic hypotheses explaining it are well‐documented in the literature, encompassing evolutionary processes (e.g., limited dispersal and specialization to narrow climatic niches), climatic conditions (e.g., decreasing temperature and oxygen availability), and the species‐area relationship (e.g., Mccain 2007; Stevens et al. 2019). A unique aspect of our study landscape is that the natural wildfire typically starts on mountaintops and then spreads across the lowland plains (Damasceno‐Junior et al. 2021). Consequently, these dynamics may influence habitat selection in the long term, potentially favoring conditions that allow highly mobile species to escape and survive the fires as they move downhill. Moreover, the local progressive structural simplification of vegetation along the elevational gradient and the reduced availability of ecological resources and refugia suggest a mechanistic link to the decline in vertebrate diversity, especially within the mountaintop ecosystems known as *Campos de altitude*, which are characterized by their unique structural and compositional features (Pessi et al. 2023).

During the last sampling season, some species were no longer detected ( *Crypturellus undulatus* , *Herpailurus yagouaroundi*, *Procyon cancrivorus* , and *Guerlinguetus aestuans*). However, our failure to detect these species is concerning and warrants continuous monitoring to better understand their population dynamics (i.e., if they are locally absent after mega‐fires or if it was only a methodological failure to detect these species). Additionally, most species considered here showed a decline in naïve relative abundance after the 2020 M‐fires compared to the pre‐burn period. Some species exhibiting higher postburn abundance are generalists, such as *A. ameiva* and *D. novemcinctus* , which may be more resilient in heavily disturbed environments. In contrast, species like giant otters and herons experienced significant declines. Giant otters are semi‐aquatic, meaning they are affected in two types of habitats. In terrestrial environments, they are impacted by the loss of wetland and riparian forest habitats due to fire (Batista et al. 2024). In aquatic environments, they are affected by habitat alterations (such as increased light levels and water temperature, reduced oxygen concentration, and toxic sedimentation) and the consequent decline in food resources (i.e., fish) (Beakes et al. 2014; Cooper et al. 2015; Whitney et al. 2015; Gomez Isaza et al. 2022). Some species even showed an initial increase in abundance during the first sampling campaign following the fire, only to experience a subsequent decline, underscoring the need for long‐term monitoring to assess the full impact of fire disturbances on these populations.

Lastly, the positive association between *I. iguana* and the presence of domesticated species and humans may be linked to anthropogenic food sources that support the proliferation of this species, which, due to its generalist nature, has demonstrated a notable ability to invade other regions (Rivera‐Milán and Haakonsson 2020). The positive association with forest may be related to the increased availability of cover, which reduces the species' visibility and exposure to predators, thereby lowering predation risk and improving thermoregulation (Lima and Dill 1990; Rusch et al. 2023).

Although no immediate fire‐driven changes in habitat selection were detected, indirect effects on species occupancy may become more apparent over longer timescales. Forest‐dependent species, such as *Tapirus terrestris* and *Panthera onca* , showed positive associations with tree cover, indicating that continued loss of forested areas due to recurrent fires (Tyukavina et al. 2022) could jeopardize their long‐term persistence. Furthermore, postfire habitat simplification may lead to delayed declines due to reduced prey availability or increased interspecific competition, as observed in other fire‐prone ecosystems (Doherty et al. 2022). Moreover, the effects vary across spatiotemporal scales. Tomas et al. (2021) report an apocalyptic scenario at the individual level and in the immediate term. In contrast, despite a resilient occupancy, we observe a predominant population decline over the medium term. But for how long will the species remain resilient? A simulation study has estimated that recovery from anthropogenic fires could take up to 50 years (Althoff et al. 2016). Additionally, the frequency of natural fires, historically occurring every 3–6 years, has increased by 2 to 3 times due to human‐induced fires (Pivello et al. 2021). Given our findings, we can consider the forest areas in the Serra da Amolar region an important fauna refuge that should be prioritized in conservation planning and firefighting, especially to protect vulnerable species in the long term. We also recommend that future studies focus on extinction, colonization, and population density analyses, and where possible, prioritize data collection closer to the occurrence of fires and over extended timescales to improve the sensitivity of analyses to fire impacts.

## Author Contributions

**Natasha Grosch Loureiro:** conceptualization (equal), data curation (equal), formal analysis (equal), investigation (lead), software (equal), writing – original draft (lead), writing – review and editing (lead). **Diego Francis Passos Viana:** investigation (equal), methodology (equal), writing – review and editing (equal). **Ciar D. Noble:** conceptualization (equal), formal analysis (equal), software (equal), writing – review and editing (equal). **Grasiela Edith de Oliveira Porfírio:** investigation (equal), methodology (equal), writing – review and editing (equal). **Wener Hugo Arruda Moreno:** data curation (equal), investigation (equal), methodology (equal), writing – review and editing (equal). **Mariana Alves Carvalho Queiroz:** investigation (equal), methodology (equal), writing – review and editing (equal). **Geovani Vinco Tonolli:** investigation (equal), methodology (equal), writing – review and editing (equal). **Carlos A. Peres:** conceptualization (equal), supervision (equal), writing – review and editing (equal). **Hugo Fernandes‐Ferreira:** conceptualization (equal), supervision (lead), writing – review and editing (equal).

## Funding

This work was supported by Coordenação de Aperfeiçoamento de Pessoal de Nível Superior—CAPES (Grants 88887.701740/2022‐00, 88887.971414/2024‐00, 88887.143948/2025‐00).

## Conflicts of Interest

The authors declare no conflicts of interest.

## Supporting information


**Figure S1:** Wildfires map in the Pantanal (red areas) at both the biome‐wide and local scale (detailed study area; in gray), during the years 2019, 2020, 2021, and 2022 (MAPBIOMAS 2023a).
**Figure S2:** Land use and land cover map at the local level (study area, Serra do Amolar, Pantanal, Brazil), in the years 2019, 2020, 2021, and 2022 (MAPBIOMAS 2023b). Forest formation (in dark green) represents forest habitat. It is possible to see spatial shifts in vegetation from 2020 onwards, especially the conversion of wetland areas into grasslands.
**Figure S3:** Topographic map showing the altitudinal variation found across the study area (Serra do Amolar; hatched), which ranged between 0 (in light green) and 975 m (in white) above sea level (USGS (U.S. Geological Survey) 2023).
**Figure S4:** Map of open water bodies showing water occurrence across the study area, based on data from the Global Water Surface (Pekel et al. 2016).
**Figure S5:** Correlation analysis showing significant correlations (correlation coefficients > 0.7) between different buffer sizes of the same predictor variable. No significant correlations were found among the various predictor variables across different buffers.
**Figure S6:** Raw relative abundance (i.e., independent photos per 100 CTNs) over the three sampling periods before the major western Pantanal fires (2019–20) and in the aftermath of major fires (2020–21 and 2021–22). The red dashed line represents the 2020 megafire event. The panels display species plots as follows: A = *Iguana iguana* , B = *Cariama cristata* , C = *Crax fasciolata* , D = *Leptotila verreauxi* , E = *Ortalis canicollis* , F = *Tigrisoma lineatum* , G = *Cerdocyon thous* , H = *Dasyprocta azarae* , I = *Eira barbara* , J = *Hydrochoerus hydrochaeris* , and K = *Leopardus pardalis* .
**Figure S7:** Raw relative abundance (i.e., independent photos per 100 CTNs) over the three sampling periods before the major western Pantanal fires (2019–20) and in the aftermath of major fires (2020–21 and 2021–22). The red dashed line represents the 2020 megafire event. Panels display species plots as follows: A = *Mazama americana* , B = *Subulo gouazoubira*, C = *Myrmecophaga tridactyla* , D = *Nasua nasua* , E = *Panthera onca* , F = *Dicotyles tajacu*, G = *Priodontes maximus* , H = *Puma concolor* , I = *Tapirus terrestris* , and J = *Tayassu pecari* .
**Figure S8:** Camera‐trap images of the species with the most interesting results. A = *Leopardus pardalis* , B = *Tapirus terrestris* , C = *Mazama americana* , D = *Panthera onca* , E = *Hydrochoerus hydrochaeris* , F = *Crax fasciolata* , G = *Pteronura brasiliensis* , H = *Iguana iguana* , J = *Tigrisoma lineatum* , K = *Ardea cocoi* , L = *Dasypus novemcinctus* , M = *Tupinambis* sp.
**Figure S9:** Mean beta coefficients for species‐level occupancy at Serra do Amolar, Mato Grosso do Sul, Brazil, inserted within the Pantanal wetlands, between 2019 and 22. Gray bars represent relationships where the 75% Bayesian credible interval (BCI) includes zero. The panels display species plots as follows: A = *Pipile cumanensis* , B = *Aramides cajaneus*, C = *Ardea cocoi* , D = *Cariama cristata* , E = *Crypturellus undulatus*, F = *Leptotila verreauxi* , G = *Ortalis canicollis* , H = *Theristicus caudatus* , and I = *Ameiva ameiva* .
**Figure S10:** Mean beta coefficients for species‐level occupancy between 2019 and 2022 at Serra do Amolar, Mato Grosso do Sul, Brazil, embedded within the Pantanal wetlands. Gray bars represent relationships where the 75% Bayesian credible intervals (BCIs) include zero. Orange bars indicate that the 75% BCI excludes zero, but the 95% BCI still includes zero. The panels display graphs of the species: A = *Cerdocyon thous* , B = *Dasyprocta azarae* , C = *Dasypus novemcinctus* , D = *Didelphis albiventris* , E = *Eira barbara* , F = *Euphractus sexcinctus* , G = *Herpailurus yaguaroundi*, H = *Subulo gouazoubira*, I = *Myrmecophaga tridactyla* , J = *Nasua nasua* , K = *Dycotiles tajacu*, L = *Priodontes maximus* , M = *Procyon cancrivorus* , *N* = *Pteronura brasiliensis* , O = *Puma concolor* , *P* = *Guerlinguetus aestuans*, and Q = *Tayassu pecari*.
**Table S1:** Model selection for detection variables (i.e., temperature, precipitation, number of cameras, and number of days deployed) for each major taxon (i.e., birds, mammals, and reptiles), based on the Watanabe‐Akaike Information Criterion (WAIC) and the effective number of parameters (pD). Rows in bold show the selected model for each taxon, between the five best models for each. Full modes include the means of the four detection variables. N Cameras = number of cameras, Days Cameras = days within the 7‐day session that the camera stayed in the field, Temperature = average temperature within each session, Precipitation = average precipitation within each session.
**Table S2:** Model selection according to buffer size (i.e., 100, 500, 1000, and 2000 m) for landscape variables (i.e., percentage of burned area and forest) for each taxon (i.e., birds, mammals, and reptiles), based on the Watanabe‐Akaike Information Criterion (WAIC), Expected Log Predictive Density (elpd), and the effective number of parameters (pD). The bold rows show the selected buffer size for each taxa's modeling.
**Table S3:** Raw relative abundance (i.e., records per 100 Camera Trap Nights) compared between the first period before the 2020 megafires and the last period after the fires. The difference between these periods is expressed as a percentage, with positive numbers representing an increase, and negative numbers representing a decline.

## Data Availability

The data that support the findings of this study are available on Dryad, DOI: https://doi.org/10.5061/dryad.80gb5mm5n.
