# Supplementary material for: Living in the Pyrocene: Assemblage‐Wide Terrestrial Vertebrate Responses to Mega‐Fires in the Pantanal Wetlands
Source: Ecol Evol. 2026 Aug 15;16(8):e74130. doi: 10.1002/ece3.74130 (PMC13476761; doi:10.1002/ece3.74130)
Supplement: Supplementary file 1 [file ECE3-16-e74130-s001.docx]

**Supplementary Information (SI)**

**Living in the Pyrocene: assemblage-wide terrestrial vertebrate responses to mega-fires in the Pantanal Wetlands**

Natasha Grosch Loureiro^1^, Diego F. P. Viana^2^, Ciar D. Noble^3^, Grasiela E. O. Porfírio^2^, Wener H. A. Moreno^2^, Mariana A. C. Queiroz^2^, Geovani V. Tonolli^2^, *Carlos A. Peres^3,4^, Hugo Fernandes-Ferreira^1,5^

^1^Universidade Federal do Ceará, Centro de Ciências, Departamento de Biologia, Programa de Pós-graduação em Sistemática, Uso e Conservação da Biodiversidade, Avenida Mister Hull, S/N, Fortaleza, Ceará, CEP 60455-760, Brazil.

^2^ Instituto Homem Pantaneiro - IHP, Corumbá, MS, CEP 79300-010, Brazil.

^3^ School of Environmental Sciences, University of East Anglia, Norwich NR4 7TJ, UK.

^4^ Instituto Juruá, Manaus, Brazil.

^5^ Universidade Estadual do Ceará, Laboratório de Conservação de Vertebrados Terrestres (Converte), Rua José de Queiroz Pessoa 2554, Quixadá, Ceará, CEP 63902-098 Brazil.

***Corresponding author email -** c.peres@uea.ac.uk

**Figure S01.** Wildfires map in the Pantanal (red areas) at both the biome-wide and local scale (detailed study area; in gray), during the years 2019, 2020, 2021, and 2022 (MapBiomas, 2023a).

**Figure S02.** Land use and land cover map at the local level (study area, Serra do Amolar, Pantanal, Brazil), in the years 2019, 2020, 2021, and 2022 (MapBiomas, 2023b). Forest formation (in dark green) represents forest habitat.It is possible to see spatial shifts in vegetation from 2020 onwards, especially the conversion of wetland areas into grasslands.

**Figure S03.** Topographic map showing the altitudinal variation found across the study area (Serra do Amolar; hatched), which ranged between 0 (in light green) and 975 meters (in white) above sea level (USGS, 2023).

**Figure S04.** Map of open water bodies showing water occurrence across the study area, based on data from the Global Water Surface (Pekel et al., 2016).

**Figure S05.** Correlation analysis showing significant correlations (correlation coefficients > 0.7) between different buffer sizes of the same predictor variable. No significant correlations were found among the various predictor variables across different buffers.

**Figure S06.** Raw relative abundance (i.e., independent photos per 100 CTNs) over the three sampling periods before the major western Pantanal fires (2019-20) and in the aftermath of major fires (2020-21 and 2021-22). The red dashed line represents the 2020 megafire event. The panels display species plots as follows: A = *Iguana iguana*, B = *Cariama cristata*, C = *Crax fasciolata*, D = *Leptotila verreauxi*, E = *Ortalis canicollis*, F = *Tigrisoma lineatum*, G = *Cerdocyon thous*, H = *Dasyprocta azarae*, I = *Eira barbara*, J = *Hydrochoerus hydrochaeris*, and K = *Leopardus pardalis*.

**Figure S07.** Raw relative abundance (i.e., independent photos per 100 CTNs) over the three sampling periods before the major western Pantanal fires (2019-20) and in the aftermath of major fires (2020-21 and 2021-22). The red dashed line represents the 2020 megafire event. Panels display species plots as follows: A = *Mazama americana*, B = *Subulo gouazoubira*, C = *Myrmecophaga tridactyla*, D = *Nasua nasua*, E = *Panthera onca*, F = *Dicotyles tajacu*, G = *Priodontes maximus*, H = *Puma concolor*, I = *Tapirus terrestris*, and J = *Tayassu pecari*.

**Figure S08.** Camera-trap images of the species with the most interesting results. A = *Leopardus pardalis*, B = *Tapirus terrestris*, C = *Mazama americana*, D = Panthera onca, E = Hydrochoerus hydrochaeris, F = Crax fasciolata, G = Pteronura brasiliensis, H = Iguana iguana, J = Tigrisoma lineatum, K = Ardea cocoi, L = Dasypus novemcinctus, M = *Tupinambis* sp.

**Figure S09.** Mean beta coefficients for species-level occupancy at Serra do Amolar, Mato Grosso do Sul, Brazil, inserted within the Pantanal wetlands, between 2019-22. Gray bars represent relationships where the 75% Bayesian credible interval (BCI) includes zero. The panels display species plots as follows: A = *Pipile cumanensis*, B = *Aramides cajaneus*, C = *Ardea cocoi*, D = *Cariama cristata*, E = *Crypturellus undulatus,* F = *Leptotila verreauxi*, G = *Ortalis canicollis*, H = *Theristicus caudatus*, and I = *Ameiva ameiva*.

**Figure S10.** Mean beta coefficients for species-level occupancy between 2019 and 2022 at Serra do Amolar, Mato Grosso do Sul, Brazil, embedded within the Pantanal wetlands. Gray bars represent relationships where the 75% Bayesian credible intervals (BCIs) include zero. Orange bars indicate that the 75% BCI excludes zero, but the 95% BCI still includes zero. The panels display graphs of the species: A = *Cerdocyon thous*, B = *Dasyprocta azarae*, C = *Dasypus novemcinctus*, D = *Didelphis albiventris*, E = *Eira barbara*, F = *Euphractus sexcinctus*, G = *Herpailurus yaguaroundi*, H = *Subulo gouazoubira*, I = *Myrmecophaga tridactyla*, J = *Nasua nasua*, K = *Dycotiles tajacu*, L = *Priodontes maximus*, M = *Procyon cancrivorus*, N = *Pteronura brasiliensis*, O = *Puma concolor*, P = *Guerlinguetus aestuans*, and Q = *Tayassu pecari.*

**Table S1.** Model selection for detection variables (i.e., temperature, precipitation, number of cameras, and number of days deployed) for each major taxon (i.e., birds, mammals, and reptiles), based on the Watanabe-Akaike Information Criterion (WAIC) and the effective number of parameters (pD). Rows in bold show the selected model for each taxon, between the five best models for each. Full modes include the means of the four detection variables. N Cameras = number of cameras, Days Cameras = days within the 7-day session that the camera stayed in the field, Temperature = average temperature within each session, Precipitation = average precipitation within each session.

| **Model** | **Taxa** | **elpd** | **pD** | **WAIC** |
| --- | --- | --- | --- | --- |
| **Psi(.), p (Days Cameras + Temperature +** **N Cameras)** | **Birds** | **-49.10901** | **15.26629** | **128.75062** |
| Full | Birds | -46.38081 | 18.26407 | 129.28975 |
| Psi(.), p (Days Cameras + N Cameras) | Birds | -51.17999 | 13.87830 | 130.11659 |
| Psi(.), p (N Cameras) | Birds | -51.41862 | 13.73850 | 130.31423 |
| Psi(.), p (Days Cameras + Precipitation + N Cameras) | Birds | -49.55227 | 15.97524 | 131.05501 |
| **Psi(.), p (Days Cameras + Temperature + N Cameras)** | **Mammals** | **-105.54750** | **38.58555** | **288.26609** |
| Psi(.), p (Days Cameras + Precipitation + N Cameras) | Mammals | -109.55846 | 36.85807 | 292.83307 |
| Psi(.), p (N Cameras) | Mammals | -113.48426 | 33.79847 | 294.56547 |
| Psi(.), p (Days Cameras + N Cameras) | Mammals | -111.30100 | 36.06388 | 294.72977 |
| Full | Mammals | -107.17329 | 40.53568 | 295.41794 |
| **Full** | **Reptiles** | **-15.986568** | **9.102946** | **50.179028** |
| Psi(.), p (Days Cameras + Precipitation) | Reptiles | -17.842057 | 8.775295 | 53.23704 |
| Psi(.), p (Precipitation) | Reptiles | -17.81185 | 8.87016 | 53.36402 |
| Psi(.), p (Days Cameras + Precipitation + N Cameras) | Reptiles | -17.778372 | 8.945849 | 53.448444 |
| Psi(.), p (Days Cameras + Temperature + Precipitation) | Reptiles | -17.415021 | 9.491943 | 53.813927 |

**Table S2.** Model selection according to buffer size (i.e., 100, 500, 1000, and 2000 meters) for landscape variables (i.e., percentage of burned area and forest) for each taxon (i.e., birds, mammals, and reptiles), based on the Watanabe-Akaike Information Criterion (WAIC), Expected Log Predictive Density (elpd), and the effective number of parameters (pD). The bold rows show the selected buffer size for each taxa's modeling.

| **Buffer size (meters)** | **Taxa** | **elpd** | **pD** | **WAIC** |
| --- | --- | --- | --- | --- |
| 100 | Birds | -47.33025 | 16.27350 | 127.20750 |
| 500 | Birds | -46.11485 | 14.33840 | 120.90651 |
| 1000 | Birds | 145.65632 | 14.63968 | 120.59199 |
| **2000** | **Birds** | **-45.80798** | **13.88704** | **119.39005** |
| 100 | Mammals | -102.22102 | 36.95482 | 278.35168 |
| 500 | Mammals | -100.68108 | 36.43478 | 274.23171 |
| **1000** | **Mammals** | **-99.75530** | **36.17579** | **271.86219** |
| 2000 | Mammals | -100.72655 | 36.82912 | 275.11133 |
| **100** | **Reptiles** | **-10.583428** | **9.968958** | **41.104773** |
| 500 | Reptiles | -11.70376 | 11.58082 | 46.56917 |
| 1000 | Reptiles | -12.39967 | 12.74554 | 50.29042 |
| 2000 | Reptiles | -15.54115 | 15.16918 | 61.42066 |

**Table S3.** Raw relative abundance (i.e., records per 100 Camera Trap Nights) compared between the first period before the 2020 megafires and the last period after the fires. The difference between these periods is expressed as a percentage, with positive numbers representing an increase, and negative numbers representing a decline.

|  | **Species** | **SamplingPeriod 1** | **Sampling Period 3** | **Difference (%)** |  |  |
| --- | --- | --- | --- | --- | --- | --- |
|  | *Pteronura brasiliensis* | 2.72 | 1.32 | -51.58 |  |  |
|  | *Ardea cocoi* | 3.17 | 1.64 | -48.36 |  |  |
|  | *Hydrochoerus hydrochaeris* | 2.85 | 1.85 | -35 |  |  |
|  | *Tayassu pecari* | 2.44 | 1.65 | -32.23 |  |  |
|  | *Iguana iguana* | 2.8 | 2.07 | -26.16 |  |  |
|  | *Tigrisoma lineatum* | 3.57 | 2.78 | -22.22 |  |  |
|  | *Ortalis canicollis* | 2.28 | 1.95 | -14.57 |  |  |
|  | *Eira barbara* | 1.81 | 1.61 | -10.75 |  |  |
|  | *Subulo gouazoubira* | 2.05 | 1.92 | -6.57 |  |  |
|  | *Crax fasciolata* | 1.79 | 1.69 | -5.06 |  |  |
|  | *Cerdocyon thous* | 1.69 | 1.61 | -4.92 |  |  |
|  | *Dicotyles tajacu* | 1.77 | 1.69 | -4.44 |  |  |
|  | *Tapirus terrestris* | 1.81 | 1.74 | -3.52 |  |  |
|  | *Dasyprocta azarae* | 1.84 | 1.78 | -3.27 |  |  |
|  | *Priodontes maximus* | 1.72 | 1.69 | -1.41 |  |  |
| *Nasua nasua* | | 1.68 | 1.67 | -0.71 |  |  |
| *Mazama americana* | | 1.84 | 1.85 | 0.79 |  |  |
| *Leopardus pardalis* | | 1.82 | 1.84 | 1.21 |  |  |
| *Leptotila verreauxi* | | 2.28 | 2.37 | 3.7 |  |  |
| *Puma concolor* | | 1.76 | 1.83 | 3.94 |  |  |
| *Panthera onca* | | 1.69 | 1.87 | 10.67 |  |  |
| *Cariama cristata* | | 1.5 | 1.69 | 13.33 |  |  |
| *Myrmecophaga tridactyla* | | 2.14 | 2.5 | 16.88 |  |  |
| *Dasypus novemcinctus* | | 1.98 | 3.23 | 63.05 |  |  |
| *Ameiva ameiva* | | 1.69 | 3.23 | 90.68 |  |  |
